# Detection of Acromegaly From Facial Images Using Machine Learning: A Comparison With Clinical Experts

**DOI:** 10.1210/jendso/bvaf203

**Published:** 2025-12-10

**Authors:** Konstantina Vouzouneraki, Erik Ylipää, Tommy Olsson, Katarina Berinder, Charlotte Höybye, Maria Petersson, Sophie Bensing, Anna-Karin Åkerman, Henrik Borg, Bertil Ekman, Jonas Robért, Britt Edén Engström, Oskar Ragnarsson, Pia Burman, Per Dahlqvist

**Affiliations:** Department of Public Health and Clinical Medicine, Umeå University, Umeå SE-901 87, Sweden; Department of Science and Technology, AIDA Data Hub, Linköping University, Linköping SE-58185, Sweden; Department of Public Health and Clinical Medicine, Umeå University, Umeå SE-901 87, Sweden; Department of Endocrinology, Karolinska University Hospital and Department of Molecular Medicine and Surgery, Karolinska Institutet, Stockholm SE-171 76, Sweden; Department of Endocrinology, Karolinska University Hospital and Department of Molecular Medicine and Surgery, Karolinska Institutet, Stockholm SE-171 76, Sweden; Department of Endocrinology, Karolinska University Hospital and Department of Molecular Medicine and Surgery, Karolinska Institutet, Stockholm SE-171 76, Sweden; Department of Endocrinology, Karolinska University Hospital and Department of Molecular Medicine and Surgery, Karolinska Institutet, Stockholm SE-171 76, Sweden; Department of Internal Medicine, Örebro University Hospital and Faculty of Health and Medical Sciences, Örebro University, Örebro SE-701 82, Sweden; Department of Endocrinology, Skåne University Hospital, Lund University, Malmö SE-205 02, Sweden; Departments of Endocrinology in Linköping and Norrköping and Department of Health, Medicine and Caring Sciences, Linköping University Linköping SE-58185, Sweden; Departments of Endocrinology in Linköping and Norrköping and Department of Health, Medicine and Caring Sciences, Linköping University Linköping SE-58185, Sweden; Department of Medical Sciences, Endocrinology and Mineral Metabolism, Uppsala University, Uppsala University Hospital, Uppsala SE-751 85, Sweden; Department of Endocrinology, Sahlgrenska University Hospital, Gothenburg SE-413 45, Sweden; Department of Internal Medicine and Clinical Nutrition, Institute of Medicine at Sahlgrenska Academy, University of Gothenburg, Gothenburg SE-413 45, Sweden; Wallenberg Centre for Molecular and Translational Medicine, Institute of Medicine, University of Gothenburg, Gothenburg SE-413 45, Sweden; Department of Endocrinology, Skåne University Hospital, Lund University, Malmö SE-205 02, Sweden; Department of Public Health and Clinical Medicine, Umeå University, Umeå SE-901 87, Sweden

**Keywords:** acromegaly, diagnostic delay, screening, face classification, face photographs, machine learning, deep learning

## Abstract

**Context:**

Substantial diagnostic delay in acromegaly contributes to increased morbidity and mortality. Screening attempts in high-risk groups have yielded few positive cases, underscoring the need for simple and precise prescreening methods.

**Objective:**

Machine-learning analysis of facial images shows promise for acromegaly detection but requires validation in larger, well-characterized cohorts using robust machine-learning frameworks as performed in this study.

**Methods:**

Facial images from different angles were collected via smartphone from 155 acromegaly patients (79% biochemically controlled) and 153 matched controls at all Swedish university hospitals. Six machine-learning models were trained to distinguish acromegaly from control images, including 3 deep neural networks pretrained on diverse image datasets (ImageNet models: ResNet50, InceptionV2, and DenseNet121) and 1 network pretrained specifically on facial images (FaRL). Model performance was compared to assessment by 12 experienced endocrinologists.

**Results:**

The diagnostic accuracy of the FaRL-based model was superior to all ImageNet models and matched the accuracy of human experts (area under the receiver operating characteristic curve 0.89 for both) with similar specificity (0.87 vs 0.93) but higher sensitivity (0.82 vs 0.66). Classification agreement between the best machine-learning model (FaRL) and human experts was 86% for true negatives and 60% for true positives. Machine-learning models and human experts both showed greater sensitivity in identifying acromegaly in male patients.

**Conclusion:**

A deep learning model pretrained on facial features (FaRL) can detect acromegaly from standard photographs with accuracy comparable to that of expert endocrinologists. This supports the feasibility of face analysis as a screening tool for acromegaly.

Acromegaly is a rare disorder characterized by slow progression and subtle early symptoms, often resulting in delayed diagnosis [1, 2]. Even in contemporary series, the median diagnostic delay is approximately 5 to 6 years—a latency that continues to contribute to excess morbidity, mortality, and reduced quality of life [1, 3]. Despite guideline recommendations to increase clinical vigilance, early recognition remains challenging due to the nonspecific nature of symptoms and rarity of the disease [4, 5]. The estimated likelihood of a Swedish general practitioner encountering a patient with undiagnosed acromegaly per year of practice is less than 1% based on an incidence of 4.0/million/year among adults [6]. Efforts to screen for acromegaly in populations with associated comorbidities such as sleep apnea, carpal tunnel syndrome, and diabetes using serum insulin-like growth factor 1, have yielded very low positive rates (0%-0.35%) [7‐9].

Facial changes are a hallmark feature of acromegaly and often the key to diagnosis [2, 10, 11]. Typical changes in acromegaly are wider face, enlarged mandible, enlarged nose, and thicker lips, with males often exhibiting more pronounced features [12, 13]. While some soft tissue changes may regress with treatment, skeletal alterations generally persist [12, 14, 15].

Several previous studies have explored the potential of automated facial image analysis to detect acromegaly but a validated screening tool for clinical use to identify patients with acromegaly is still lacking. Although some machine-learning models have reported high diagnostic accuracy, concerns remain about their generalizability due to small samples, use of unrepresentative or unmatched control images, and lack of ethnic diversity [16‐21]. Moreover, few prior studies have benchmarked the outcome of different models against clinical experts as a reliability measure [17, 21, 22].

Given the persistent diagnostic delay and central role of facial features in acromegaly, we aimed to develop and validate a machine learning–based prediction tool for acromegaly detection from facial photographs using various deep learning architectures on a national cohort with acromegaly and matched controls. We also compared its diagnostic performance to that of experienced endocrinologists.

## Materials and Methods

### Participants

Most of the patients and controls in this study were also included in a previously published, parallel, voice analysis study by our group [23]. Briefly, patients with acromegaly, both newly and previously diagnosed, were invited to participate in the study at all 7 university hospitals in Sweden between February 2021 and May 2023. For each patient included with acromegaly, 1 control individual from the same center matched for sex and age (±5 years) was recruited either from the staff or patients in the endocrine clinic with diagnoses not affecting facial characteristics. The only exclusion criterion for this study was inability to read or understand the study material. However, a few patients were not invited to participate if excluded due to an exclusion criterion in the previous voice analysis study [23].

### Procedure

**Figure 1.**
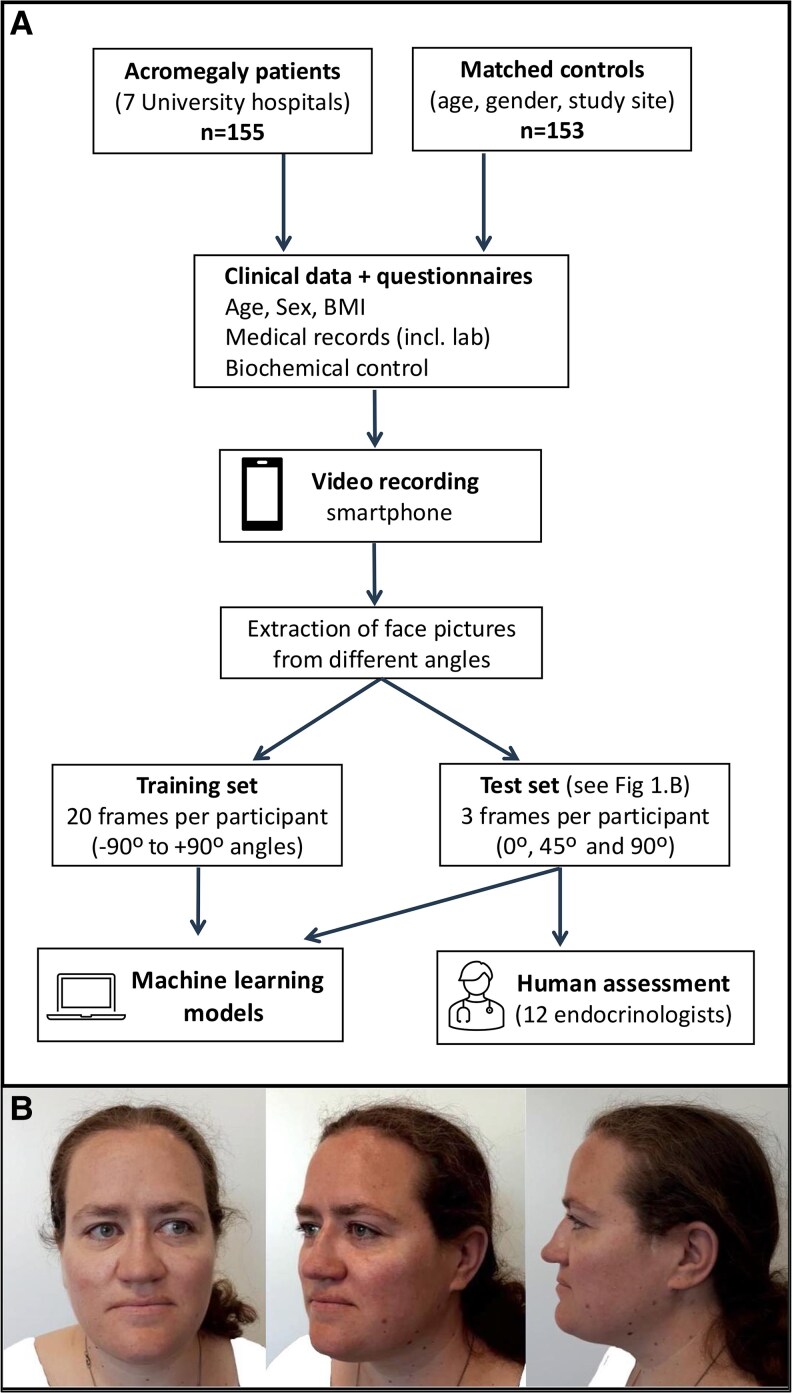
A, Flowchart showing the inclusion of patients and matched controls and data processing used for the machine-learning model setup and endocrinologists' assessment. B, Examples of the 3 images (0°, 45°, and 90°) from a patient with acromegaly extracted from the video recordings and used in the test set of the machine-learning models and human expert endocrinologists. Clothing was masked with white pixels. Published with the patient's consent. BMI, body mass index; incl., including.

At the study visit, specialized research nurses made a standardized video recording of the participant's face using a customized smartphone (Samsung Galaxy A40). Video recordings of patients and controls were made with participants sitting on a chair rotating from −90° to +90° in a room with a white background and light toward the participant's face. Clinical data were collected including date of diagnosis and treatments of acromegaly, duration of acromegalic symptoms, biochemical control as assessed by the treating physician (based on clinical, radiological, and biochemical data), duration of control, size of pituitary adenoma at diagnosis, laboratory data, anthropometric data, and current medications, as described in our previous publication of the same cohort (Fig. 1) [23].

### Image Data Preprocessing

From the recorded videos, images from multiple angles were extracted using a preprocessing pipeline in which the videos were first manually cropped to include the individual’s head. The rotation around the body's vertical axis (yaw) was estimated from the frames of the images using the SixDRepNet360 model [24]. From the yaw-estimated images, 2 sets of images were extracted from each participant's video recording: 1) 20 images from different yaw angles ranging from −90° to +90° used for training the machine-learning models (image set 1); and 2) 3 images per participant (0°, 45°, and 90°) used for testing the machine-learning models and the human experts (image set 2) (see Fig. 1). Due to the risk of clothing-related correlations, as many controls were health-care workers wearing uniforms, clothing in all images was masked with white pixels using the model face-parsing PyTorch, a version of BiSeNet (Bilateral Segmentation Network) performing semantic segmentation fine-tuned for face parsing [25, 26]. The masks of image set 2 (used in the test set) were further manually adjusted to ensure that all clothing was fully masked and thus avoid spurious associations due to clothing. The masks of image set 1 (used in the training set) were not manually adjusted, considering that as long as the images used for testing (image set 2) do not contain the spurious association, any such false association learned during training cannot produce falsely inflated performance estimates.

### Machine-Learning Models

The machine-learning models used in our study included 4 different pretrained neural networks. Three networks (ResNet50, InceptionV3, DenseNet121) were from the Torchvision framework, pretrained on the ImageNet-1K dataset, which contains more than 1.2 million diverse, real-world photographs of varied categories like animals, everyday objects, scenes, and concepts. Of these images, about 250 000 contain faces [27]. The fourth model, the Face Representation Learning (FaRL) 64-epoch model, is a pretrained model on the LAION-Face dataset consisting of 20 million images of human faces [28]. FaRL uses a contemporary transformer neural network architecture with 87-M parameters, while the other 3 models use more traditional convolutional neural networks with 26-M (ResNet50), 27-M (InceptionV3), and 8-M (DenseNet121) parameters.

The fine-tuning task was set up as a binary classification of each picture as acromegaly or control. During training, performance on a development set was tracked using the optimal area under the receiver operating characteristic curve (ROC AUC) as the criterion. The best performing model on the development set was selected as the final model for the training. For the FaRL model, an early stopping criterion was used with a patience of 5, that is, stopping training if no improvement was seen after 5 epochs, due to increased computational demands. An epoch refers to a complete pass through the training dataset leading to modification of the model's parameters. All neural networks were trained using a 2-phase training program: Initially, only the newly added prediction heads were trained (with the pretrained part frozen) and, thereafter, training of the whole network. The final model was chosen by the best performance on the development set. In total 1100 (900 Torchvision models, 200 FaRL) neural networks were trained. The code for model training is available at GitHub platform (https://github.com/eryl/acroface-submission).

The neural networks were also combined as ensembles in 2 different configurations. One combined the 3 ImageNet-pretrained models (DenseNet, ResNet, and InceptionV3), similar to the method used by Kizilgul and colleagues [20], which will be referred to as the ImageNet ensemble. The other was an extended ensemble, which included all 4 neural networks and will be referred to as ImageNet ensemble + FaRL.

### Model Selection Setup

**Figure 2.**
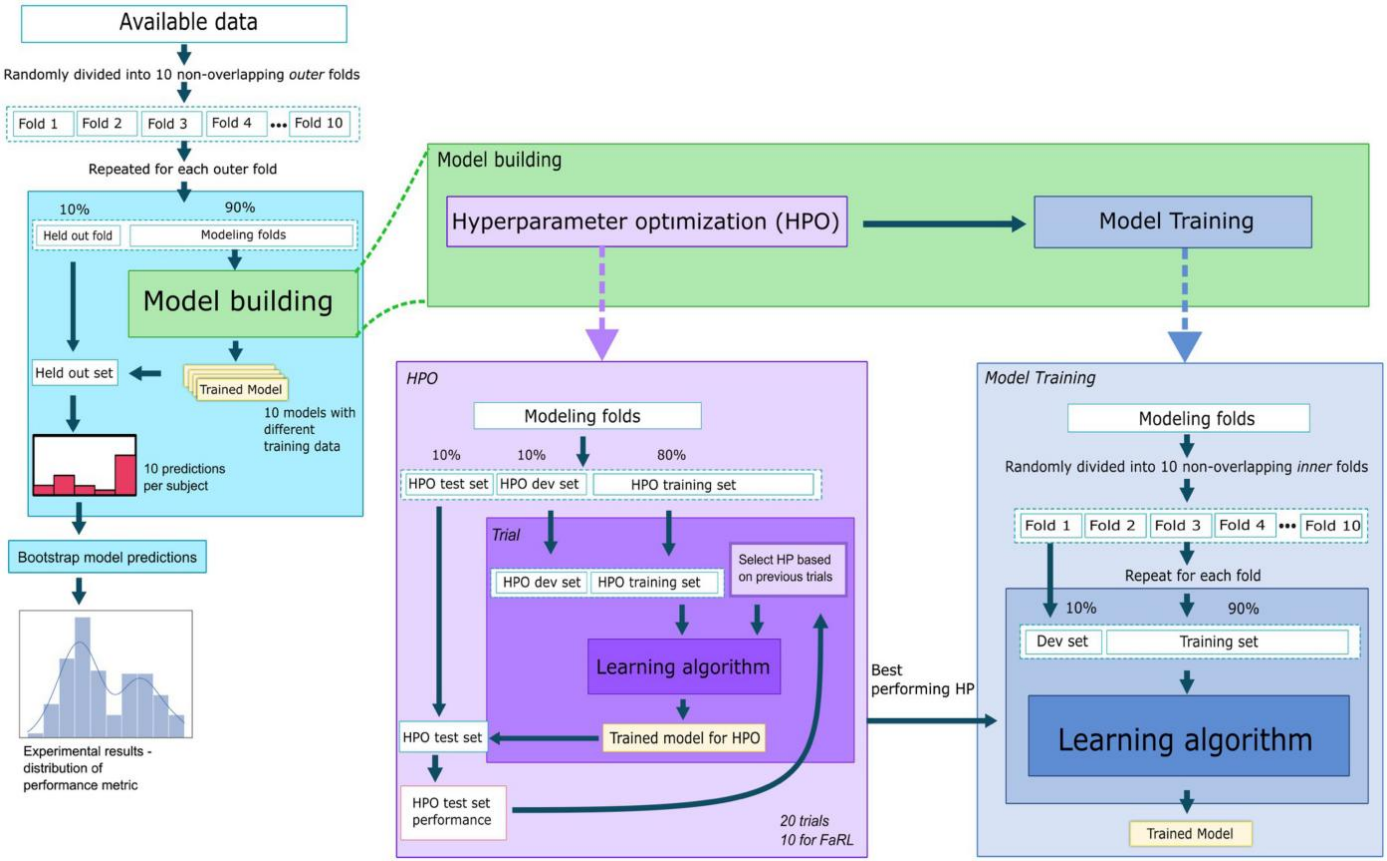
Illustration of machine-learning model development. During training, 10-fold cross-validation was performed on the whole dataset. The modeling folds were used for training and development, and the held-out subset represents the test set. Hyperparameter search included optimization of hyperparameters based on the performance of hyperparameters on the hyperparameter test set (HPO box). The best performing hyperparameters were used in model training using 10-fold cross-validation on the modeling dataset (Model Training box), where 1 fold acted as the development set (dev set) to monitor early stopping criteria and the remaining 9 folds were used as the training set. This resulted in 10 models trained for each outer test data fold trained on different subsets of the modeling set.

All machine-learning experiments followed the same procedure for model selection. They started with an outer 10-fold cross-validation loop (Fig. 2). The data split was stratified to keep the balance of acromegaly and controls the same in different folds. Furthermore, matched acromegaly and control pairs were kept in the same fold to reduce sample bias. Care was taken to ensure all sampling was based on participants, so that images from the same video did not end up in different folds. Such an error would lead to leakage from training to test sets and significantly overestimate model performance. Each outer fold (containing 10% of the participants) was held out once to serve as the test dataset (held-out fold) for that set of experiments, leaving the remaining 90% as modeling folds (see Fig. 2). On the modeling folds, a single initial split into 3 hyperparameter optimization (HPO) sets was performed: 10% HPO test, 10% HPO development, and 80% HPO training set (see Fig. 2). Using these HPO splits, HPO was performed using the Optuna framework with 20 trials for all models except for FaRL, with 10 trials due to much higher computational demands (see Fig. 2) [29]. After automatic selection of hyperparameters, nested 10-fold cross-validation was performed on the modeling dataset, where one of the folds acted as the development set to monitor early stopping criteria and the remaining folds as the training set (see Fig. 2). Using this approach, 10 models per neural network architecture were trained with the optimal hyperparameters. This process was repeated for each outer fold. Overall, for each neural network architecture, we trained 300 models (200 for FaRL), of which 100 per architecture contributed to the final results. Each individual in the study served as a test case for 10 models (all using the same initial hyperparameters), resulting in 10 predictions per participant and neural network architecture.

### Endocrinologists' Assessment

Pictures from all 308 participants (155 acromegaly patients and 153 controls) were assessed by 12 experienced endocrinologists (co-authors P.D., P.B., T.O., O.R., K.B., C.H., M.P., S.B., A.-K.Å., H.B., B.E., B.E.E) from all Swedish university hospitals using PsychoPy, an open-source application for experiments in behavioral sciences installed on their own computers [30]. A group of 3 photographs (0°, 45°, and 90° angles; see Fig. 1) from each participant was presented in random order and the endocrinologists classified each participant as either acromegaly or control. To investigate intrarater reliability, 50 randomly chosen participants were presented twice during the session. Photographs in which endocrinologists identified the participant's face as a colleague or patient of their own were excluded from the analysis.

### Statistics

Continuous variables are presented as median and interquartile range (IQR). Categorical variables are presented as absolute numbers and percentages.

Bootstrapping with 10 000 runs was performed to estimate the variance of the experts' predictions with 95% CIs. For each bootstrap run, each participant’s annotations by all experts (coded 0 for control and 1 for acromegaly) were bootstrapped and the mean was used as the “ensemble” predictions. Binary performance metrics were calculated on each bootstrap sample using a discretization threshold of 0.5 or more for positive predictions (meaning that at least 50% of the experts annotated a positive result, ie, acromegaly). ROC AUC was calculated on the bootstrapped means, not the discretized ensemble predictions.

To make predictions of the machine-learning models comparable to human experts' binary decision (acromegaly or control), the model's probability scores were binarized using a threshold. The threshold was selected independently for each model to maximize the Youden's J statistic (sensitivity + specificity – 1) on its development dataset. The Youden's J statistic was chosen as a neutral decision that equally weights sensitivity and specificity. For each model and participant in its test set, the majority binary prediction of the 3 test images (set 2) was chosen as the final prediction.

The discretized predictions (coded 0 for control and 1 for acromegaly) of the machine-learning models were bootstrapped similarly to the experts' classifications with 10 predictions per participant (by 10 models) used in bootstraps repeated 10 000 times. These bootstraps were used to estimate 95% CIs for machine-learning performance. The same method was used regardless of whether the models were evaluated separately or as part of an ensemble.

The evaluation of machine-learning models included the calculation of sensitivity, specificity, balanced accuracy, ROC AUC, and F1 score. F1 score is the harmonic mean of precision and sensitivity. Subgroup analyses were performed based on age (3 equal groups), sex, and biochemical control of acromegaly (yes/no).

## Results

We invited 300 Swedish patients with acromegaly to participate, of whom 102 declined participation and 38 were excluded due to language difficulties (n = 19) or due to an exclusion criterion in the parallel study on voice analysis in acromegaly (n = 19) [23]. Another 5 patients with acromegaly were excluded due to technical problems during video recording. Thus, the final cohort consisted of 155 acromegaly patients (42% women) with median (IQR) age at inclusion 58 years (46-67 years) and age at acromegaly diagnosis of 44 years (33-53 years) (Table 1). Among these, 19 (12%) patients were newly diagnosed and not yet treated for acromegaly, and 14 (9%) patients were previously diagnosed and treated but considered not biochemically controlled by the treating endocrinologist. To assess representability of the study cohort, we obtained a report from the Swedish Pituitary Registry at the end of the inclusion of this study (March 31, 2023). This reported 864 patients living with acromegaly in Sweden (49% women) with a median (IQR) age of 61 years (50-72) and age at acromegaly diagnosis of 48 years (37-58). Among those with registered information on biochemical control, 20% were reported to be not biochemically controlled [31].

**Table 1.** Baseline characteristics of patients with acromegaly

All patients with acromegaly (n = 155) (denominator for missing data)	
Age at inclusion, y	58 (46-67) (n = 155)
Female	65/155 (42%)
Male	90/155 (58%)
Age at acromegaly diagnosis, y	44 (33-53) (n = 154)
Time since acromegaly diagnosis, y	10 (4-18) (n = 154)
Time from acromegaly symptoms to diagnosis, y	5 (2-10) (n = 143)
Pituitary adenoma diameter at diagnosis, mm	15 (10-20) (n = 133)
Macroadenoma (>10 mm) at diagnosis	103/136 (76%)
Biochemical control by physician assessment	122 (79%)
Duration of biochemical control, y	9 (3-14) (n = 120)

Treated patients with previously diagnosed acromegaly (n = 136)*^a^*	
Pituitary surgery	130/136 (96%)
Multiple surgeries	19/136 (14%)
Radiotherapy	42/136 (31%)
Current medical therapy	
Somatostatin analogue	39/136 (29%)
Pegvisomant	21/136 (15%)
Dopamine agonist	10/136 (7%)
Combination	13/136 (10%)
Pituitary hormone replacement	
Thyroid hormone	38/136 (28%)
Testosterone	27/136 (19%)
Cortisol	25/136 (18%)
Antidiuretic hormone	7/136 (5%)

Data are n (%) or median (interquartile range).

^*a*^Excludes 19 patients with newly diagnosed acromegaly who were treatment naive.

The control group included 153 participants (50 patients with diagnoses other than acromegaly and 103 health-care workers), 143 of whom were matched with an acromegaly patient by age (±5 years), sex, and study site. A matched control could not be recruited for 12 acromegaly patients during the study period, partly due to the pandemic and partly since facial photography discouraged some to participate. Thus, we included 10 controls without individual matching to a specific acromegaly patient, but with the prerequisite that the acromegaly and control groups did not differ in terms of age and sex (Table 2). Control participants could not be recruited for 2 patients with acromegaly.

**Table 2.** Baseline characteristics of patients with acromegaly vs controls

Characteristic	Acromegaly (n = 155)	Controls (n = 153)	*P*
Female	65 (42%)	67 (44%)	.742
Male	90 (58%)	88 (56%)	
Age, y	57.8 (46.2-67.2)	57.4 (46.4-66.4)	.795
Weight, kg	89.8 (78-105)	79.1 (69-92)	<.001
Height, cm	177 (169-186)	175 (167-182)	.044
BMI	28.1 (25.3-31.2)	25.3 (23.1-28.8)	<.001

Data are n (%) or median (interquartile range).

Missing data: age (n = 1), height (n = 4), weight (n = 6), and BMI (n = 6).

Abbreviation: BMI, body mass index.

### Endocrinologists' Assessment

The experienced endocrinologists correctly identified pictures of acromegaly patients with a median sensitivity of 0.62 and specificity of 0.87, with a large variation between the experts (Supplementary Table S1) [32]. The balanced accuracy of individual endocrinologists ranged from 0.72 to 0.79 with a median of 0.75 (see Supplementary Table S1) [32]. We observed a trend for higher sensitivity and specificity in the assessment of photographs of male compared to female participants (see Supplementary Table S1) [32]. The median intrarater reliability was high with Cohen's κ 0.85 (range, 0.70-0.93). The ensemble of expert classifications for each participant, as described in the statistics section, showed a sensitivity of 0.66, specificity of 0.93, and ROC AUC 0.89 (Table 3).

**Table 3.** Bootstrap precision metrics for all prediction models and endocrinologists' assessment

Predictor	Sensitivity	Specificity	ROC AUC	Balanced accuracy	F1
Endocrinologists	0.66 (0.62-0.70)	0.93 (0.90-0.96)	0.89 (0.88-0.91)	0.80 (0.77-0.82)	0.76 (0.73-0.80)
FaRL	0.82 (0.79-0.84)	0.87 (0.84-0.90)	0.89 (0.88-0.90)	0.80 (0.77-0.82)	0.84 (0.82-0.86)
ImageNet ensemble	0.75 (0.72-0.77)	0.80 (0.77-0.84)	0.85 (0.84-0.86)	0.78 (0.76-0.80)	0.77 (0.75-0.79)
ImageNet ensemble + FaRL	0.78 (0.75-0.81)	0.85 (0.82-0.88)	0.88 (0.88-0.89)	0.82 (0.80-0.84)	0.81 (0.79-0.83)
DenseNet	0.74 (0.70-0.77)	0.78 (0.75-0.81)	0.83 (0.82-0.85)	0.76 (0.73-0.78)	0.75 (0.73-0.78)
Inceptionv3	0.74 (0.71-0.77)	0.76 (0.72-0.80)	0.82 (0.81-0.84)	0.75 (0.72-0.78)	0.75 (0.72-0.78)
ResNet	0.76 (0.73-0.79)	0.72 (0.69-0.76)	0.82 (0.80-0.83)	0.74 (0.72-0.77)	0.75 (0.72-0.77)

Data are presented as mean (95% CI). ImageNet ensemble combines DenseNet, Inception V3, and ResNet.

Abbreviation: ROC AUC, area under the receiver operating characteristic curve.

### Machine-Learning Models

**Figure 3.**
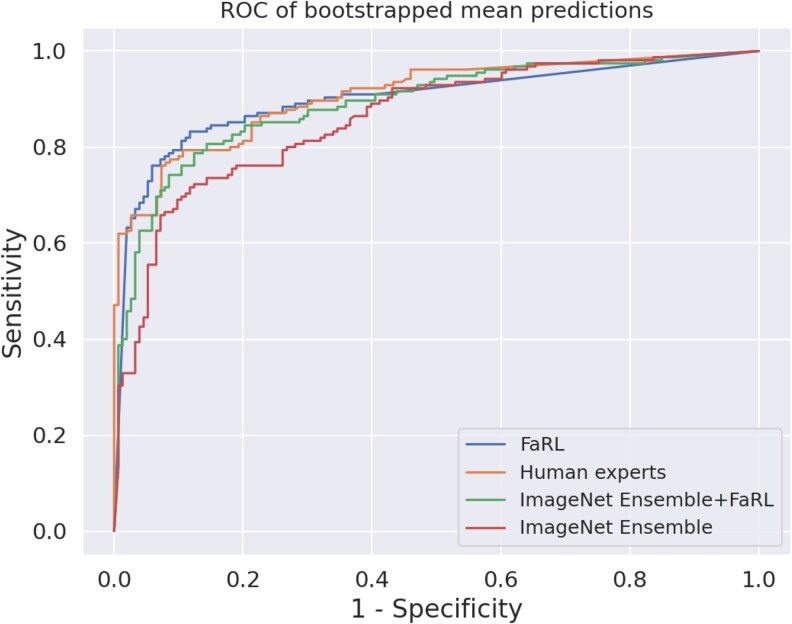
ROC AUCs of bootstrapped mean predictions of ImageNet ensemble, FaRL, ImageNet ensemble + FaRL, and human expert ensemble. Each machine-learning model's discretization threshold was set to the optimal cutoff according to Youden's index. For human expert assessment, a discretization threshold of 0.5 or greater was used. ROC AUC, area under the receiver operating characteristic curve.

The pretrained models from ImageNet (ResNet, InceptionV2, and DenseNet) showed similar sensitivity (0.74-0.76), specificity (0.72-0.78), balanced accuracy (0.74-0.76), and ROC AUC (0.82-0.83) (see Table 3). All 3 of these models appeared to have higher sensitivity but lower specificity compared to the human experts, which resulted in a larger ROC AUC for the human experts (0.89) (see Table 3). The ensemble of these 3 models (ImageNet ensemble) displayed better performance in all metrics than any of the individual models' independent predictions (see Table 3). We also developed a model based on FaRL, a machine-learning model pretrained on human faces [28]. The FaRL-based model showed better performance than all the individual ImageNet models and the ensemble based on these (see Table 3, Fig. 3). Compared to the ensemble of 12 expert endocrinologists, the FaRL model presented a higher sensitivity (0.82 vs 0.66) with a comparable specificity (0.87 vs 0.93) and identical compound performance metrics (ROC AUC 0.89, balanced accuracy 0.89) (see Table 3 and Fig. 3). An ensemble based on the ImageNet ensemble and the FaRL model was not superior to the FaRL model alone (see Table 3 and Fig. 3).

### Level of Agreement

**Figure 4.**
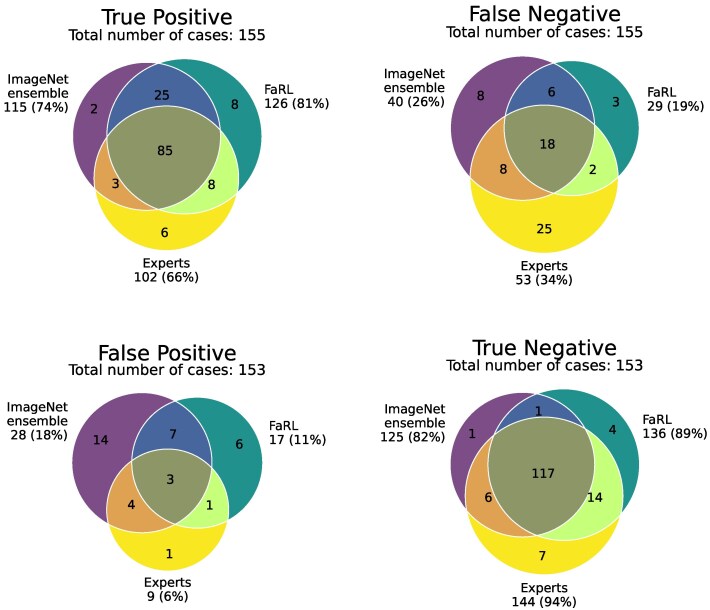
Venn diagram illustrating the overlap of true-positive, false-negative, false-positive, and true-negative predictions of the ImageNet ensemble, FaRL, and experts' assessment.

The agreement of predictions between the model based on FaRL, the ImageNet ensemble, and the endocrinologists' ensemble is shown in Fig. 4. Of the 155 patients with acromegaly, 137 were correctly classified by at least 1 of the aforementioned models (true positive), whereas 18 were missed by all 3 (false negative). Only 85 patients were correctly classified by all 3. FaRL correctly classified 126 of 155 (81%) patients with acromegaly. Of the remaining 29 patients (15 men, 4 newly diagnosed) missed by FaRL, 20 patients (8 men, 3 newly diagnosed) were also misclassified by the endocrinologists' ensemble. Conversely, the endocrinologists' ensemble missed 53 patients with acromegaly, of which 33 were correctly diagnosed by the FaRL algorithm. Agreement for control individuals was generally high across all methods, with the endocrinologists' ensemble demonstrating slightly higher specificity than FaRL (94% vs 89%) (see Fig. 4).

### Performance in Subgroups

To assess whether diagnostic performance of image classification varied according to sex, age, or biochemical disease status, we conducted subgroup analyses based on sex, biochemical activity (active vs controlled disease), and 3 equal-size age groups (23-51, 52-64, and 65-86 years). Sex-specific analyses revealed higher sensitivity for detecting acromegaly in men compared to women both for the machine-learning models and the human experts, while specificity was similar between sexes (Supplementary Table S2) [32]. The FaRL model and the endocrinologists' ensemble showed a trend toward higher sensitivity and ROC AUC in the age group 52 to 64 years with minimal change in specificity (see Supplementary Table S2) [32]. In the youngest group (age 23-51 years), FaRL demonstrated higher sensitivity than the endocrinologists' ensemble (0.73 vs 0.58). No statistically significant differences in performance metrics were observed between patients with biochemically active and controlled disease for either the machine-learning models or the human assessors (see Supplementary Table S2) [32].

## Discussion

This is the first study evaluating a machine-learning model pretrained exclusively on facial images (FaRL) to detect acromegaly from smartphone photographs. Compared with 12 experienced endocrinologists, the FaRL-based model achieved a higher sensitivity with only slightly lower specificity, resulting in comparable overall accuracy.

The performance of the ImageNet-based models in our study was modest (see Table 3) compared to the study by Kizilgul and colleagues [20], which showed an exceptional ROC AUC of 0.96 to 0.99 using the same pretrained ImageNet networks and similar study design and data acquisition process [20]. Several factors may account for this discrepancy. Our study included all available contemporary patients with acromegaly, including also cases with mild facial phenotypes, some of whom were not classified as acromegaly by expert review. Controls were carefully matched for age, sex, and study site to minimize bias. Rashwan et al [21] recently reported a 3-dimensional reconstruction approach based on past printed frontal pictures provided by patients and unmatched controls. The faces were classified as having mild, moderate, or severe acromegalic features by 9 participating endocrinologists and the machine-learning model predictions were compared against expert grading rather than a confirmed acromegaly diagnosis [21]. However, as illustrated in our Venn diagrams (see Fig. 4), expert judgment based solely on facial features is not flawless and cannot substitute for a verified diagnosis [21]. Differences in cohort composition, phenotypic spectrum, and image acquisition likely account for the varying reported performance across studies [20, 21].

Earlier studies of face classification systems for acromegaly relied on shallow models without deep learning with smaller datasets, web-sourced images with overt acromegalic facial features, or unmatched and demographically limited control groups [16‐19], thereby limiting generalizability. For instance, Miller et al [16] used male-only controls at age 30 to 50 years, while the control images in the study by Gencturk et al [18] were collected from online databases dominated by women and children. Kong and colleagues [19] included a larger cohort that included web-sourced images of patients with acromegaly and control images partly acquired from datasets developed for facial beauty perception [33], potentially introducing selection bias. In contrast, our study use a larger acromegaly cohort with standardized photographs and demographically matched controls, increasing validity and clinical relevance.

FaRL and ImageNet models both demonstrated higher sensitivity in men, aligning with prior studies and human experts [17, 21]. This likely reflects more prominent craniofacial changes in men [12, 13]. Biological sex differences in disease expression, such as lower insulin-like growth factor 1 levels and subtler phenotypes in women, may contribute to the longer diagnostic delays in women [34‐36]. Interestingly, diagnostic performance was similar in patients with active vs biochemically controlled acromegaly. This may seem somewhat surprising since acromegaly treatment can partially reverse facial soft tissue hypertrophy [37]. However, skeletal craniofacial changes persist even after biochemical control of acromegaly [12, 14], maintaining distinctive differences from controls.

Key strengths of this study include the prospective, standardized image acquisition, a large multicenter cohort, and inclusion of matched controls. The use of a face-specific pretrained model (FaRL) enabled strong performance despite limited data volume, achieving an ROC AUC of 0.89, on par with expert review but with higher sensitivity, which is a desirable feature in screening applications. Notably, our case-to-control ratio (∼1:1 in the study design) is far higher than the true prevalence of acromegaly in any screening scenario. We therefore focused on reporting sensitivity, specificity, balanced accuracy, and ROC AUC, which are metrics that remain stable irrespective of disease prevalence.

Limitations include the predominance of patients in biochemical remission (79%), which means that many patients had been treated and might have less pronounced soft tissue features. Additionally, the study population was predominantly of Swedish/European ancestry (∼89% of acromegaly patients and 92% of controls), so our model's performance may not directly extrapolate to other ethnic groups. Thus, further validation in larger, unselected cohorts is an essential next step to develop a clinical diagnostic tool for acromegaly. The endocrinologists assessed the photographs as they would do in a real-life clinical setting without specific instructions on favoring specificity or sensitivity. This may have contributed to differences in the diagnostic threshold used by each expert. However, the inclusion of 12 endocrinologists from different regions with high levels of experience aimed to minimize this variation and provide a fair comparison to the machine-learning models.

In the same cohort of acromegaly patients and controls, we have previously reported a classification model based on voice analysis using a combination of shallow machine-learning models to identify acromegaly. This showed an accuracy close to the FaRL-based face model (ROC AUC 0.84 vs 0.89) [23]. Considering that these models evaluate different parameters and were developed using very different machine-learning architectures, a future multimodal analysis of voice and face analysis could be beneficial to further enhance diagnostic accuracy.

The long-term goal of this work is to contribute to the development of a clinically applicable digital diagnostic assistant for acromegaly. Following external validation, such a tool could be used for acromegaly screening in clinics seeing patients with relevant comorbidities, for example, sleep apnea and carpal tunnel syndrome, which are often diagnosed years before acromegaly diagnosis, opening a time-window for earlier diagnosis. We envision a multimodal stepwise process in which artificial intelligence–based prescreening based both on voice and face analysis is complemented by expert review of other clinical features to enhance sensitivity and specificity. Further studies are essential to evaluate different screening strategies, the optimal sensitivity and specificity threshold, and their cost effectiveness in different high-risk populations. Finally, by making our code publicly available, we provide—for the first time in this field—a reproducible framework that enables independent validation, refinement, and future development toward a widely accessible screening tool.

### Conclusions

A facial image–based machine-learning model (FaRL) can detect acromegaly with accuracy comparable to that of experienced endocrinologists. With further training on larger, more diverse populations and external validation, this technology has the potential to become a valuable screening tool, reducing the diagnostic delay in acromegaly and consequently improving patient outcomes and quality of life [38].

## Data Availability

Anonymized data are available on request except for images due to the sensitive nature of these personal data.

## Abbreviations

BMI: body mass index
FaRL: Face Representation Learning
HPO: hyperparameter optimization
IQR: interquartile range
ROC AUC: area under the receiver operating characteristic curve
